# Cellular interplay between cardiomyocytes and non-myocytes in diabetic cardiomyopathy

**DOI:** 10.1093/cvr/cvac049

**Published:** 2022-04-07

**Authors:** Ren Jie Phang, Rebecca H Ritchie, Derek J Hausenloy, Jarmon G Lees, Shiang Y Lim

**Affiliations:** O’Brien Institute Department, St Vincent’s Institute of Medical Research, 9 Princes Street, Fitzroy, VIC 3065, Australia; Department of Surgery, University of Melbourne, Parkville, VIC 3010, Australia; School of Biosciences, University of Melbourne, Parkville, VIC 3010, Australia; Drug Discovery Biology, Monash Institute of Pharmaceutical Sciences, Parkville, VIC 3052, Australia; Department of Pharmacology, Monash University, Clayton, VIC 3800, Australia; National Heart Research Institute Singapore, National Heart Centre Singapore, Singapore, Singapore; Cardiovascular and Metabolic Disorders Programme, Duke-NUS Medical School, Singapore, Singapore; Yong Loo Lin School of Medicine, National University Singapore, Singapore, Singapore; The Hatter Cardiovascular Institute, University College London, London, UK; Cardiovascular Research Center, College of Medical and Health Sciences, Asia University, Taichung City, Taiwan; O’Brien Institute Department, St Vincent’s Institute of Medical Research, 9 Princes Street, Fitzroy, VIC 3065, Australia; Department of Medicine, University of Melbourne, Parkville, VIC 3010, Australia; O’Brien Institute Department, St Vincent’s Institute of Medical Research, 9 Princes Street, Fitzroy, VIC 3065, Australia; Department of Surgery, University of Melbourne, Parkville, VIC 3010, Australia; Drug Discovery Biology, Monash Institute of Pharmaceutical Sciences, Parkville, VIC 3052, Australia; National Heart Research Institute Singapore, National Heart Centre Singapore, Singapore, Singapore

**Keywords:** Diabetic cardiomyopathy, Cardiomyocytes, Endothelial cells, Autonomic neurons, Cardiac fibroblasts, Immune cells

## Abstract

Patients with Type 2 diabetes mellitus (T2DM) frequently exhibit a distinctive cardiac phenotype known as diabetic cardiomyopathy. Cardiac complications associated with T2DM include cardiac inflammation, hypertrophy, fibrosis, and diastolic dysfunction in the early stages of the disease, which can progress to systolic dysfunction and heart failure. Effective therapeutic options for diabetic cardiomyopathy are limited and often have conflicting results. The lack of effective treatments for diabetic cardiomyopathy is due in part, to our poor understanding of the disease development and progression, as well as a lack of robust and valid preclinical human models that can accurately recapitulate the pathophysiology of the human heart. In addition to cardiomyocytes, the heart contains a heterogeneous population of non-myocytes including fibroblasts, vascular cells, autonomic neurons, and immune cells. These cardiac non-myocytes play important roles in cardiac homeostasis and disease, yet the effect of hyperglycaemia and hyperlipidaemia on these cell types is often overlooked in preclinical models of diabetic cardiomyopathy. The advent of human-induced pluripotent stem cells provides a new paradigm in which to model diabetic cardiomyopathy as they can be differentiated into all cell types in the human heart. This review will discuss the roles of cardiac non-myocytes and their dynamic intercellular interactions in the pathogenesis of diabetic cardiomyopathy. We will also discuss the use of sodium-glucose cotransporter 2 inhibitors as a therapy for diabetic cardiomyopathy and their known impacts on non-myocytes. These developments will no doubt facilitate the discovery of novel treatment targets for preventing the onset and progression of diabetic cardiomyopathy.

## 1. Introduction

Diabetes mellitus is a growing global pandemic, representing a significant cause of morbidity and mortality around the world.^1,2^ A recent meta-analysis estimated that diabetes mellitus affected 463 million people worldwide in 2019, and the number of patients with diabetes mellitus is expected to increase to 629 million by 2045.^3^ Diabetes is a sustained condition of elevated blood glucose levels and is an umbrella term encompassing Type 1, Type 2, and gestational diabetes mellitus. Type 1 diabetes is an autoimmune-mediated disorder that results from T cell-mediated destruction of β-cells in the pancreas, leading to an insulin deficiency.^4^ Type 2 diabetes mellitus (T2DM) is a highly complex, multisystem disorder, which is characterized by overt hyperglycaemia because of insulin resistance, impaired insulin secretion, and increased hepatic glucose output.^5^ Gestational diabetes is a pregnancy complication in which women with no known risk factors and without previously diagnosed diabetes develop hyperglycaemia, and its incidence is on the rise in recent decades.^6^ Among the three types of diabetes, T2DM accounts for 85–95% of all cases.

T2DM is a complex systemic disorder that can be attributed to a combination of genetic and environmental risk factors. Risk factors such as age, increased adiposity, sedentary lifestyle, smoking, and perturbed gut microbiome have been shown to cause hyperglycaemia and hyperlipidaemia in T2DM.^7,8^ At the community level, urbanization in developed and developing countries has cultivated sedentary behaviour and an obesogenic environment with inexpensive high sugar and high-fat diets, which have fuelled the T2DM epidemic.^3^ In some cases, individuals with a specific variation in T2DM-associated genes are more susceptible to the development of T2DM. A single nucleotide polymorphism in the *TCF7L2* gene identified by a genome-wide association study, which encodes a protein that regulates insulin production, predisposes individuals to T2DM.^9,10^

## 2. Pathophysiological processes of T2DM

Blood glucose homeostasis is monitored by the pancreatic islet β-cells and regulated by insulin action on the target cells.^11^ In the insulin signalling cascade, the insulin receptor substrate-1 is a crucial docking molecule for the insulin receptor, and its activation promotes the translocation of the glucose transporter to the cell membrane.^12^ In T2DM, the functions of the insulin receptor and insulin receptor substrate-1 in glucose uptake are reduced, which is known as insulin resistance. This is mainly attributed to mitochondrial dysfunction, inflammation, ectopic lipid accumulation, and endoplasmic reticulum stress in the insulin target cells eventually leading to the development of hyperglycaemia.^1,13^ Insulin resistance-induced hyperglycaemia triggers β-cells to intensify their synthesis and secretion of insulin to overcome insulin resistance and restore glucose homeostasis.^11^ However, this compensatory hyperinsulinaemia eventually causes the pancreatic β-cells to progressively lose their capacity to produce a sufficient amount of insulin to offset the peripheral insulin resistance.^14,15^ Hence, persistent impaired insulin action, together with chronic β-cell dysfunction, marks the clinical diagnosis of overt T2DM.

Overt hyperglycaemia is the core characteristic of T2DM and a potent risk factor for diabetes-related complications in the late stage of the disease.^1^ An important group of diabetes-related complications are vascular complications, which can be further classified into microvascular and macrovascular complications.^16^ Microvascular complications comprise diabetes-related retinopathy, nephropathy, and neuropathy.^16^ T2DM patients are at 10–20 times higher risk of developing microvascular disorders,^17^ which arise from hyperglycaemia-induced thickening of capillary basement membranes.^18^ The underlying mechanism of microvascular complications includes the production of advanced glycation end-products (AGEs), activation of the polyol pathway, low-grade inflammation, and increased endoplasmic reticulum stress.^19^ On the other hand, insulin resistance and hyperglycaemia can also promote atherosclerosis and cause macrovascular complications including coronary artery disease, stroke, and peripheral vascular disease which are 2–4 times more likely in T2DM patients.^16,17^ Independent of these macrovascular complications, diabetes mellitus can cause impairments to cardiac structure and function and has long been known to increase the risk of heart failure.^20^

## 3. Diabetic cardiomyopathy

Over one million Australians have T2DM, just over half of whom will die from cardiovascular disease, and even more will have serious but non-fatal cardiovascular complications.^21^ Indeed, adults with T2DM are 2–4 times more likely to experience heart failure than adults without diabetes.^22^ Heart failure caused specifically by diabetes, in the absence of other conditions such as ischaemic heart disease and hypertension, is called diabetic cardiomyopathy, for which there are currently no effective treatments. The development of heart failure in T2DM patients is usually attributed to macrovascular disease, leading to heart failure with reduced ejection fraction (HFrEF, ejection fraction < 50%).^23^ However, the development of heart failure independent of macrovascular complications is gaining attention, known as heart failure with preserved ejection fraction (HFpEF).^24,25^ The early manifestations of diabetic cardiomyopathy include left ventricular concentric hypertrophy, increased filling pressures, and impaired diastolic function, yet ejection fraction remains >50%, which resembles the cardiac phenotype of HFpEF.^26^ This cardiac phenotype may be followed by further impairment of diastolic function and the onset of systolic dysfunction, resembling HFrEF.^26^ While patients with diabetic cardiomyopathy often present with HFpEF, differences in sex-bias and the incidence and different manifestations of hypertrophy suggest that it would be naïve to view diabetic cardiomyopathy as just another manifestation of HFpEF.^27^ Indeed, the clinical manifestations of diabetic cardiomyopathy are still not completely understood, and the mechanisms underlying the disease progression from HFpEF to HFrEF are unclear.^28,29^ This knowledge gap has impeded the development of effective treatments for diabetic cardiomyopathy. Therefore, it is important to develop a greater molecular and cellular understanding of pathophysiological phenotypes and disease progression in diabetic cardiomyopathy using clinically relevant disease models.

### 3.1 Sex differences

In non-diabetic patients, the risk of cardiovascular disease is higher in men. However, diabetes completely reverses this sex difference, and in diabetic patients the risk of cardiovascular disease is higher in women.^30–32^ Even in diabetic patients with comparable glycaemic control, an increased cardiovascular risk factor profile is reported in diabetic women relative to men.^33^ For example, the added risk of heart failure associated with diabetes is five-fold higher in diabetic women compared with just 2.4-fold higher in diabetic men.^34^ The increased risk of heart failure may be related to the heightened susceptibility of women with newly diagnosed T2DM to pathogenic remodelling of the left ventricle and reduced myocardial energy efficiency.^35^ In streptozocin-induced mouse models of diabetic cardiomyopathy, despite no differences in cardiac structure between males and females, diabetic females are more susceptible to diastolic dysfunction despite exhibiting a lower extent of hyperglycaemia than males.^36^ Cardiovascular complications including fibrosis, atherosclerosis, and endothelial dysfunction are also differentially regulated by sex in diabetes.^37^ For example, diabetic women are more prone to endothelial dysfunction,^38,39^ and several markers of endothelial dysfunction are female-specific and independent of age or body mass index including plasminogen activator inhibitor-1, intercellular adhesion molecule-1, and E-selectin levels.^40,41^ Furthermore, endothelial dysfunction and insulin resistance are associated with worse cardiovascular outcomes in women than men with Type 1 or Type 2 diabetes.^40,41^ Sex differences have also been reported in mouse models of Type 1 diabetic cardiomyopathy,^42^ and in mitochondrial fusion and fission proteins in the foetal rat heart of offspring born to diabetic mothers.^43^

## 4. Current understanding of the mechanisms underlying diabetic cardiomyopathy

**Figure 1:**
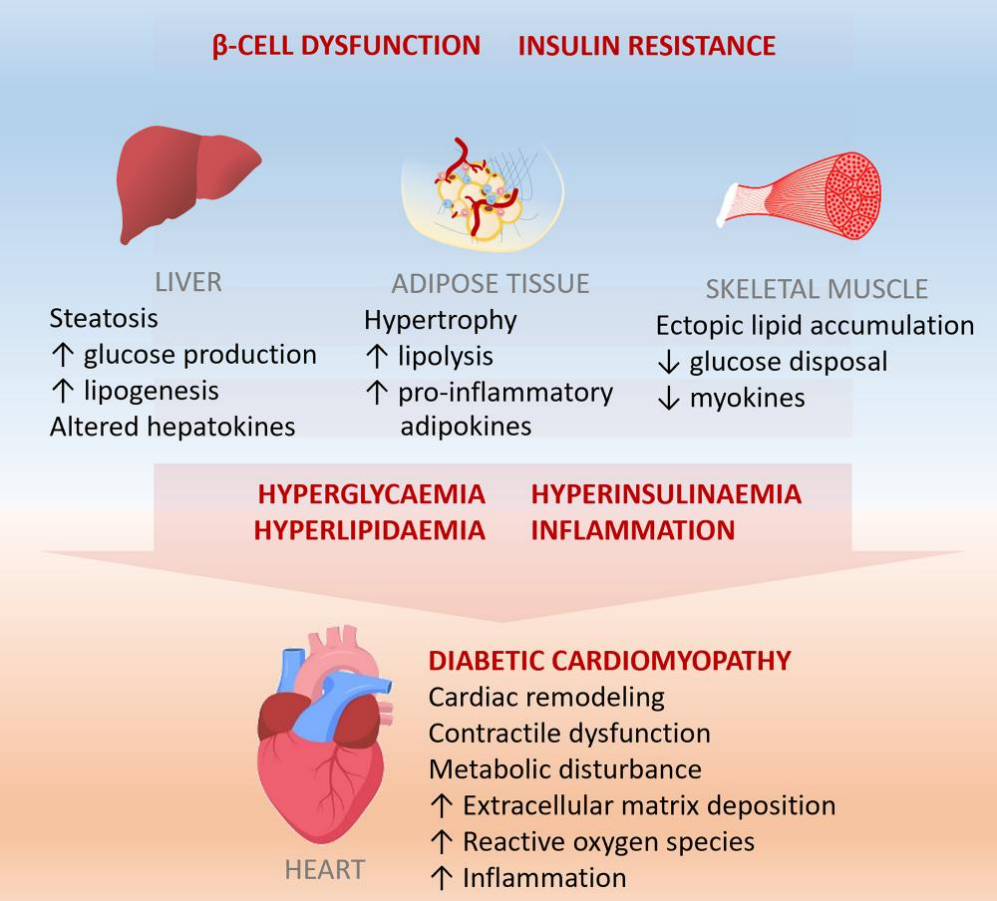
**The contribution of non-cardiac tissues to the pathogenesis of diabetic cardiomyopathy.** In diabetes, pancreatic beta-cell dysfunction and insulin resistance lead to impaired insulin-mediated glucose uptake and increased ectopic lipid accumulation in various non-cardiac tissues, including liver, adipose tissue, and skeletal muscle. Impaired insulin signalling in the liver increases hepatic glucose production, while glucose disposal in skeletal muscle is reduced, both of which contribute to systemic hyperglycaemia. To compensate for hyperglycaemia, the pancreas produces more insulin leading to hyperinsulinaemia. Furthermore, increased lipolysis of adipose tissue increases the levels of circulating free fatty acids leading to lipotoxicity and hyperlipidaemia. These pathological events result in overproduction of pro-inflammatory adipokines, dysregulation of hepatokines, and a reduction in protective myokines, all of which contributes to the pathogenesis of diabetic cardiomyopathy.

Despite a wealth of studies on diabetic cardiomyopathy, our understanding of its pathogenesis remains elusive. Driven by reduced insulin signalling and/or insulin resistance in adipose tissue, liver, and skeletal muscle, pathological changes in these non-cardiac tissues contribute to the development and progression of diabetic cardiomyopathy (Figure *1*). Additionally, abnormal cardiac extracellular matrix deposition, diabetes-induced metabolic disturbance, oxidative stress, and inflammation, can all contribute to adverse cardiac remodelling and contractile dysfunction.^5,44^

### 4.1 The role of adipose tissue in diabetic cardiomyopathy

Adipose tissues store excess energy and act as an endocrine organ to modulate the function of other organs via adipose tissue-derived signalling molecules called adipokines.^45^ Adipose tissue is classified by morphology (white, beige, or brown) and location (subcutaneous, kidneys, breast tissue, bone marrow, epicardium/visceral pericardium).^46^ Excessive expansion of adipose tissue, especially in the visceral pericardium, is a risk factor for both T2DM and diabetic cardiomyopathy.^47–51^ Obesity drives hypertrophy of white adipocytes, fibrosis, and insulin resistance, all of which lead to reduced lipid retention in the adipocytes and ectopic lipid accumulation in other organs including the liver and skeletal muscle. Ectopic lipid accumulation is observed in CCN5 knockout obese mice^52^ and high-fat diet-fed rodents,^53,54^ which also display insulin resistance and cardiac hypertrophy. In human, healthy hearts transplanted into patients with T2DM show progressive lipid accumulation in cardiomyocytes within 12 months post-transplantation.^55^ This might suggest that ectopic lipid accumulation may be one of the earliest pathologies in the development of diabetic cardiomyopathy.

The heart is surrounded by epicardial adipose tissue, which plays important roles in directing the cell fate of non-myocytes and maintaining myocardial metabolic homeostasis.^56^ With a higher basal rate of fatty acid metabolism and lipogenesis than subcutaneous and visceral adipose tissue, epicardial adipose tissue provides energy to the underlying myocardium and acts as reservoir for excess fatty acids.^51^ The pathological expansion of epicardial adipose tissue has a well-documented association with cardiometabolic diseases, including diabetic cardiomyopathy,^49,57–61^ mediated by dysregulated production of pro-inflammatory cytokines and adiponectin from epicardial adipose tissue.^46^ Epicardial adipose tissue also modulates cardiac endothelial function through its direct contact with the myocardium and the coronary arteries.^62,63^ In an *ex vivo* model of T2DM, human epicardial adipose tissue treated with diabetic conditions increased the expression of pro-inflammatory cytokines, such as TNF-α and interleukin (IL)-1β, which subsequently induced human coronary artery endothelial cells to acquire an inflammatory phenotype and reduced angiogenic potential.^64^

### 4.2 The role of liver in diabetic cardiomyopathy

Individuals with a high fatty liver index and advanced liver fibrosis are associated with an increased risk of myocardial infarction, heart failure, and cardiovascular mortality.^65–67^ Chronic liver conditions such as non-alcoholic fatty liver disease (NAFLD) can negatively affect cardiovascular system via secretion of hepatocyte-derived cytokines, known as hepatokines.^68^ For example, serum fibroblast growth factor-21 predominantly produced and secreted by hepatocytes, is positively correlated with metabolic syndrome and the severity of liver fibrosis in T2DM patients.^69,70^ The hepatokine fetuin-A has been shown to mediate crosstalk between the liver and the heart, preventing blood vessel calcification and aortic stenosis. A lower fetuin-A concentration is associated with greater risk of microvascular and macrovascular complications in T2DM patients.^71^ Interestingly, individuals with T2DM and NAFLD often present with a cardiac autonomic nervous system imbalance, displaying an increase in sympathetic tone,^72,73^ which is associated with increased blood pressure and risk of ventricular arrythmia in T2DM.^74–76^

### 4.3 The role of skeletal muscle in diabetic cardiomyopathy

The skeletal muscle is the primary site of glucose disposal, making it a pivotal player in the development of insulin resistance.^77^ Insulin resistance in skeletal muscle stems from excessive lipid accumulation following saturation of adipose tissues, which impairs insulin receptor signalling and glucose uptake. Similar to the liver and adipose tissues, skeletal muscle establishes communication with other tissues and organs via secreted soluble proteins known as myokines such as musclin, myostatin, IL-6, IL-13, IL-15, and irisin.^78,79^ Physical inactivity has been shown to alter the secreted myokine profile, establishing a firm link between a sedentary lifestyle and several metabolic disorders.^80–82^ The therapeutic effects of myokines in diabetic cardiomyopathy have received some attention. In streptozotocin-treated mice, the overexpression of the myokine osteocrin prevented cardiomyocyte apoptosis and myocardial fibrosis by restoring cardioprotective proteasomal activity.^83^ The myokine irisin has gained increasing attention with respect to its role in the development of T2DM and diabetic cardiomyopathy.^84^ Circulating levels of irisin have been shown to be reduced in newly diagnosed diabetic patients^85^ and in T2DM *db/db* mice.^86^ Excitingly, restoring the irisin levels with either intramyocardial injection of adenovirus encoding irisin or treatment with recombinant human irisin effectively attenuated cardiac diastolic dysfunction and adverse structural remodelling in *db/db* mice.^86^*In vitro* rat cardiomyocytes treated with diabetic culture conditions lose expression of irisin, while overexpression of irisin alleviates the acquired inflammatory response, reduces oxidative stress and cardiomyocyte apoptosis, and improves mitochondrial function.^86,87^ Collectively, these studies highlighted the cardioprotective effect of myokine irisin in the setting of diabetes.

### 4.4 Abnormal cardiac extracellular matrix deposition

Hyperglycaemia and insulin resistance promote cardiac fibrosis. Patients with diabetic cardiomyopathy usually present with left ventricular hypertrophy and perivascular fibrosis.^88–90^ Hyperglycaemia induces overproduction of extracellular matrix proteins such as collagens from cardiac fibroblasts.^91^ Cardiac fibroblasts also play a physiological role in maintaining extracellular matrix homeostasis through balancing the activities of matrix metalloproteinases and tissue inhibitors of metalloproteinases.^92^ Matrix metalloproteinases induce the degradation of the extracellular matrix, but their activity is attenuated under systemic hyperglycaemia causing the overaccumulation of extracellular matrices.^93^ This is attributed to hyperglycaemia-mediated promotion of the non-enzymatic formation of AGEs, which can cross-link with the extracellular matrices and increase their resistance to the proteolytic action of matrix metalloproteinases.^93^ AGEs can also promote the differentiation of fibroblasts to myofibroblasts, which secrete pro-fibrotic cytokines and promote excessive extracellular matrix deposition.^94^ The pro-fibrotic activity of fibroblasts and myofibroblasts is further exacerbated by growth factors such as transforming growth factor β-1 (TGFβ-1), connective tissue growth factor, and various ILs, all of which are up-regulated in the diabetic myocardium.^94^ This combination results in excessive deposition of extracellular matrix and contributes to increased cardiac stiffness and reduced cardiac compliance, which are the classical hallmarks of the early stage of diabetic cardiomyopathy.^44,95^ Indeed, the degree of extracellular matrix deposition has been shown to be correlated with mortality and hospitalization for heart failure in patients with T2DM.^96^

### 4.5 Diabetes-induced impaired cardiac metabolism

The heart has a high metabolic rate relative to other tissues.^97^ The majority of energy in the form of adenosine triphosphate (ATP) in cardiomyocytes is produced via mitochondrial oxidation of substrates such as fatty acids (60–70%), glucose (20%), and lactate (10%).^98^ The ability of cardiomyocytes to utilize a selection of substrates for ATP production is known as metabolic flexibility; this is impaired in diabetic cardiomyopathy.^99^ The reduced insulin signalling and reduced glucose uptake in cardiomyocytes shift metabolism from glucose to fatty acid oxidation.^100^ In addition, suppression of insulin activity induces the activity of lipases in adipose tissue and the secretion of very-low-density lipoprotein in the liver, resulting in an increased level of free fatty acids in the circulation.^101^ These free fatty acids can be taken up by the liver and converted into ketone bodies, which provides additional metabolic fuel for extrahepatic tissues, including the heart. While studies have reported increased ketone body utilization in failing diabetic hearts as a compensatory mechanism for metabolic inflexibility,^102,103^ a recent study by Brahma *et al*.^104^ has demonstrated contrasting results, in which diabetic-induced hyperglycaemia suppressed myocardial ketolytic capacity and ketone body catabolism. Nevertheless, the net increase and accumulation of ketone bodies in the circulation increase the risk of diabetic ketoacidosis.^105^

The high levels of circulating free fatty acids can also increase the activity of peroxisome proliferator-activated receptors on cardiomyocytes, specifically the alpha subtype, which promote the expression of proteins involved in fatty acid uptake and oxidation such as fatty acids translocase, carnitine palmitoyltransferase-1, and long-chain acyl coenzyme A dehydrogenase.^106^ The adenine nucleotide transporter that transports ATP from the mitochondria to the cytosol has been shown to be inhibited by long-chain acyl coenzyme A derivatives and as a consequence, impairs myocardial contractility.^107–109^ Excessive uptake of fatty acids by the cardiomyocytes promotes the futile recycling of the lipid intermediates as a protective mechanism towards lipotoxicity.^110^ However, when the delivery of fatty acids exceeds the capacity of fatty acid oxidation these un-oxidized fatty acids can be directed into lipid metabolism pathways that synthesis triacylglycerols, ceramides, and diacylglycerols.^111^ Although triacylglycerols have a cardioprotective effect by preventing the excessive generation of reactive lipid intermediates, ceramides and diacylglycerols can activate signalling pathways leading to the development of cardiac lipotoxicity.^112^ In essence, alongside pre-existing insulin resistance, cardiomyocytes subjected to a hyperlipidaemic environment exhibit impaired metabolic flexibility, favouring beta oxidation of fatty acids and the production of reactive lipid intermediates.

### 4.6 Cardiac oxidative stress

Oxidative stress, the result of imbalanced generation and scavenging of reactive oxygen species (ROS) and reactive nitrogen species is another mechanism in the development of diabetic cardiomyopathy.^106^ In T2DM, the primary source of ROS in cardiomyocytes is superoxide produced from up-regulation of nicotinamide adenine dinucleotide phosphate (NADPH) oxidase, mitochondrial dysfunction, and uncoupling of nitric oxide (NO) synthase.^113,114^ Additionally, the endogenous antioxidant system is impaired in T2DM, which further exacerbates cardiac oxidative stress. For example, the expression of nuclear factor erythroid 2-related factor 2 (Nrf2), which is required to initiate the antioxidant response, is down-regulated in high-fat diet-induced T2DM mouse and rat models.^115,116^ The overproduction of ROS also activates multiple pathological signalling cascades including protein kinase C and c-Jun N-terminal kinase pathways, which create a detrimental feedforward loop inducing further ROS generation.^117,118^ Peroxynitrite is a potent oxidant formed through a reaction between superoxide and NO.^119^ The formation of peroxynitrite can directly degrade myofibrillar protein and DNA, and reduce NO bioavailability, which itself is implicated in the pathophysiology of diabetic cardiomyopathy.^120^ Finally, apart from damaging proteins and DNA, the accumulation of ROS in cardiomyocytes also initiates a maladaptive inflammatory response, detailed below.

### 4.7 Cardiac inflammation

T2DM is now recognized as an inflammatory disorder.^121^ Several *in vitro* and *in vivo* diabetic models have reported increased expression of pro-inflammatory factors such as tumour necrosis factor-α, IL-1β, IL-6, and NLR Family Pyrin Domain Containing 3 (NLRP3) inflammasomes in cardiomyocytes, all of which correlate with pathological cardiac remodelling such as fibrosis and hypertrophy.^87,122–124^ Cardiac inflammation in T2DM is in part mediated by ROS-induced activation of NLRP3 inflammasomes, which can lead to a cascade of signalling events that initiate cardiomyocyte apoptosis and myocardial fibrosis.^27^ Apart from NLRP3 inflammation, circulating AGEs and pro-inflammatory cytokines can activate other pro-inflammatory pathways in cardiomyocytes such as nuclear factor kappa-B expression and lipoxygenases which contribute to the development of diabetic cardiomyopathy.^125–127^ Tumour necrosis factor-α and IL-1β have been shown to increase Ca^2+^ leakage from cardiomyocyte sarcoplasmic reticula contributing to depressed systolic Ca^2+^ transients and resulting in cardiac contractile dysfunction and arrhythmia.^128^ Collectively, the inflammatory responses induced by the systemic and local changes during T2DM are diverse, and the pro-inflammatory pathways are often related and can contribute to the pathophysiology of diabetic cardiomyopathy by promoting myocardial remodelling and impairing cardiac function.

## 5. The involvement of cardiac non-myocytes in the pathogenesis of diabetic cardiomyopathy

**Figure 2:**
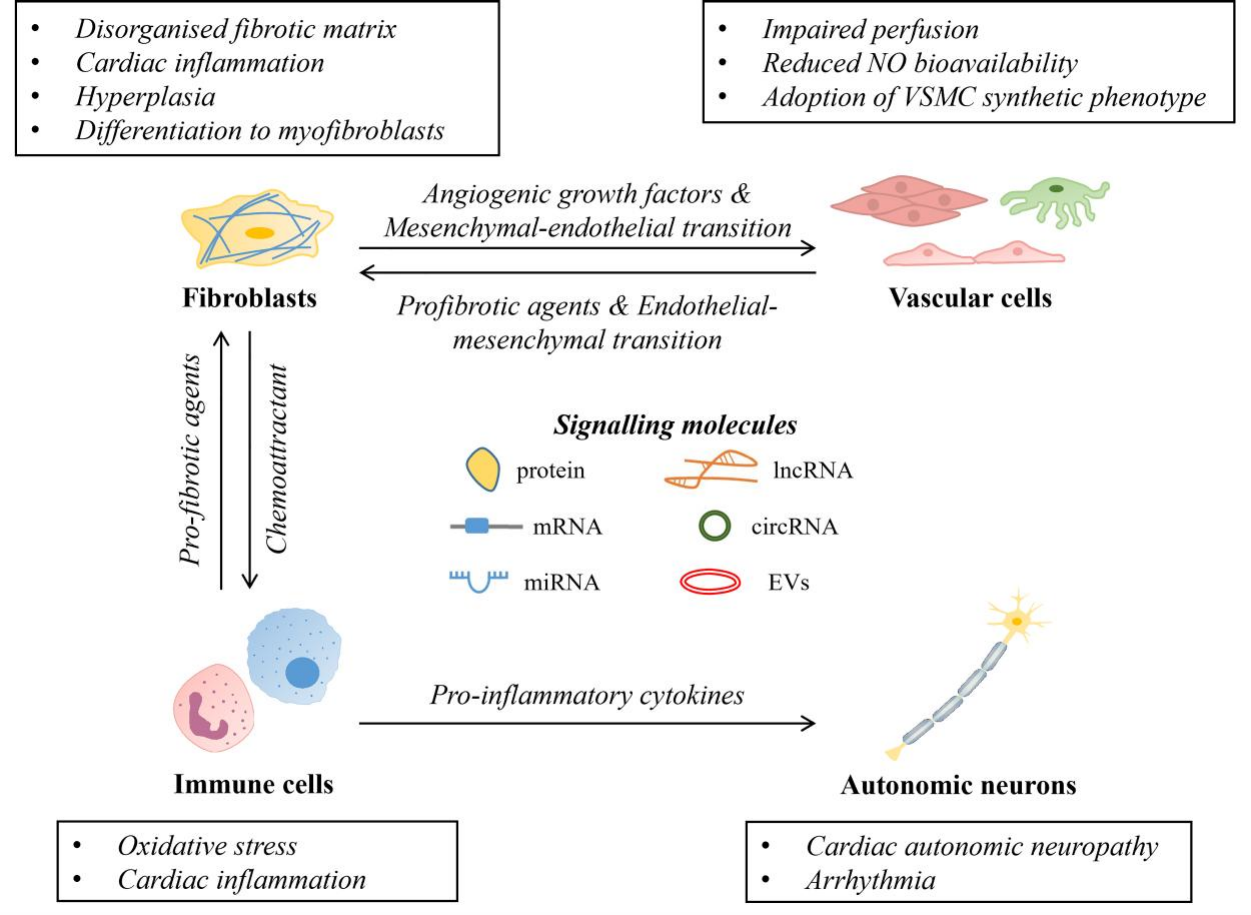
**Cellular interactions among non-myocytes in the heart and their pathological phenotypes in T2DM.** Non-myocytes populations play important roles in maintaining healthy cardiac function and promoting cardiomyopathy under diabetic conditions. Cardiac fibroblasts receive pro-fibrotic factors from immune and vascular cells to regulate the dynamics of the extracellular matrix. Conversely, cardiac fibroblasts can induce angiogenesis and infiltration of immune cells to evoke an inflammatory response via secretion of angiogenic growth factors and chemoattractants, respectively. The function of autonomic nervous system is tightly linked with the activity of the immune cells, where the modulation of neuronal activity can be achieved by pro-inflammatory cytokines. Under diabetic conditions, these intercellular interactions can be exacerbated resulting in maladaptive cellular phenotype (highlighted in boxes) and contribute to the pathogenesis of diabetic cardiomyopathy. Signalling molecules include proteins, EVs, messenger RNA (mRNA), microRNA (miRNA), long non-coding RNA (lncRNA), and circular RNA (circRNA).

Hyperglycaemia, hyperlipidaemia, and systemic changes such as increased circulating pro-inflammatory cytokines, AGEs, and ROS are the drivers for maladaptive signalling pathways in cardiomyocytes, contributing to the development of diabetic cardiomyopathy.^27,129^ However, the pathophysiology of diabetic cardiomyopathy cannot be attributed to the pathological changes of the cardiomyocytes alone. Indeed, the heart is a complex 3D structure with heterogeneous cell populations including cardiomyocytes, cardiac fibroblasts, coronary vasculature, autonomic neurons, and immune cells.^130^ The cardiac non-myocyte cell populations play an important role in regulating electrical, chemical, and biomechanical signalling in the heart and form an extensive network of intercellular communication to maintain cardiac homeostasis, as well as orchestrate the pathogenesis of diabetic cardiomyopathy (*Figure 2*). Indeed, microvascular complications and neurocardiac dysfunction are often reported in T2DM patients, suggesting the involvement of non-myocyte cell populations in the disease. However, the effect of T2DM on cardiac non-myocytes such as the cardiac vasculature, autonomic neuronal function, and its consequences remain poorly understood.^131,132^

### 5.1 The role of cardiac fibroblasts in diabetic cardiomyopathy

Cardiac fibroblasts represent ∼20% of the non-cardiomyocytes in the heart and are responsible for extracellular matrix deposition, maintenance, and remodelling.^133^ The myocardial extracellular matrix is vital in maintaining healthy cardiac structure and function by serving as a scaffold for the myocytes and non-myocytes populations. This scaffold allows transmission of electrical conduction between cardiomyocytes as well as transduction of mechanical forces throughout the myocardium and can prevent injury resulting from over-extension.^94,134^

The number of cardiac fibroblasts in the mouse heart doubles as a result of T2DM.^135^ In response to hyperglycaemia, cardiac fibroblasts up-regulate and remodel cardiac extracellular matrix components, causing fibrosis and contractile dysfunction.^136^ Impaired left ventricle compliance and diastolic dysfunction is largely attributed to increased matrix accumulation in ∼50% of T2DM patients.^137^ Hyperglycaemia has been shown to increase collagen production in neonatal and adult animal and human cardiac fibroblasts *in vitro* and *in vivo*. This contributes to exaggerated extracellular matrix deposition and can disrupt electrophysiological communication between cardiomyocytes.^134,138–143^ Moreover, increased fibrosis within the perivascular regions could form a physical barrier to oxygen and nutrient diffusion and subsequently reduce their availability to the myocardium causing focal ischaemia.^144,145^ Excessive matrix synthesis is largely driven by myofibroblasts in T2DM for which several hyperglycaemia-modulated pathways have been identified that might promote the activation of a pro-fibrotic cardiac fibroblast phenotype. Detailed molecular mechanisms of diabetic fibrosis have been recently reviewed.^134^

In addition to extracellular matrix modulation, cardiac fibroblasts can also influence cardiac electrical signalling and conduction through the expression of gap junctions, ryanodine receptors, and ion channels such as voltage-gated potassium channel and voltage-dependent calcium channel Ca_v_1.2.^128,146,147^ Although cardiac fibroblasts are non-excitable cells, they exhibit high membrane resistance and are able to conduct electrical signals.^94^ Cardiac fibroblasts can electrically couple to each other and to adjacent cardiomyocytes via gap junctional connexins (Cx40, Cx43, and Cx45) to modulate their electrophysiological properties. Alteration of these gap junctional communications can lead to arrhythmia and has been implicated in various pathological conditions including diabetic cardiomyopathy.^94,148^ Moreover, abnormal calcium signalling increases the susceptibility to cardiac arrhythmia and mitochondrial calcium handling is impaired in the diabetic heart, partly attributed to oxidative stress-induced dysfunction of the ryanodine receptors and SERCA2 protein.^128,149,150^ However, whether calcium handling protein function and ion channels in cardiac fibroblasts and myofibroblasts are also altered in diabetic conditions and contribute to diabetic cardiomyopathy remains unclear.

Cardiac fibroblasts can also interact with endothelial cells to regulate angiogenesis and contribute to vascular remodelling following cardiac injury.^94^ Fibroblasts have been shown to interact with endothelial cells indirectly via secretion of pro-angiogenic growth factors such as fibroblast growth factor and vascular endothelial growth factor, and anti-angiogenic pigment epithelium-derived growth factor.^94,151^ Cardiac fibroblasts could also directly contribute to cardiac angiogenesis by undergoing mesenchymal-to-endothelial transition and adopt an endothelial cell fate as demonstrated in subjects with ischaemic heart disease.^152^ Whether this phenomenon is also observed in the setting of diabetic cardiomyopathy, however, remains to be investigated. Furthermore, cardiac fibroblasts are the main source of pro-inflammatory cytokines such as IL-1β following injury and have been shown to promote cardiac infiltration of inflammatory cells through secretion of the chemokine CXCL10 and contribute to the cardiac inflammation in diabetes.^94,153,154^

### 5.2 The role of coronary vasculature in diabetic cardiomyopathy

The coronary vasculature is crucial for maintaining perfusion to the myocardium. Within the coronary vasculature, endothelial cells form a continuous semipermeable biological membrane that regulates the exchange of oxygen, nutrients, and waste between the circulatory system and the myocardium.^155^ Endothelial cells also provide energy substrates for neighbouring cardiomyocytes.^156^ Under physiological conditions, endothelial cells primarily utilize ATP generated from glycolysis, whereas fatty acids are primarily used to synthesize nucleotides or transported to the cardiomyocytes.^156^ However, in hyperglycaemic conditions, endothelial cells initiate a protective mechanism to prevent the entry of glucose into cardiomyocytes by reducing the expression of glucose transporter-1 at the interface between the endothelial cells and the cardiomyocytes. This, in turn, leads to the accumulation of intracellular glucose in endothelial cells, where glucose is shunted into alternative pathways such as the polyol and hexosamine biosynthesis pathways to reduce glycolytic flux.^157^ The net result is the production of ROS and AGEs that promote endothelial cell dysfunction.^158^

With their close proximity to the cardiomyocytes, any dysfunction in endothelial cells can have a direct impact on myocardial function and potentially contribute to the progression of diabetic cardiomyopathy. Indeed, T2DM patients without obstructive coronary artery disease often experience substantial impairment of coronary microvascular function caused by hyperglycaemia-induced ROS and protein kinase C activation in endothelial cells.^159^ The hallmark of endothelial dysfunction has reduced NO bioavailability. Similar to cardiomyocytes, hyperglycaemia can activate NADPH oxidase and uncouple endothelial NO synthase (eNOS) in endothelial cells, resulting in oxidative stress and reduced NO bioavailability, respectively.^119,160,161^ In addition, the uncoupling of eNOS causes its isomeric switch from NO-producing eNOS dimers to a superoxide-producing eNOS monomer, further contributing to oxidative stress.^119^ The reduction of NO bioavailability is also associated with the development of diastolic dysfunction through up-regulation of fibrotic signalling and phosphorylation of the contractile protein titin, resulting in increased myocardial stiffness and cardiac hypertrophy.^162^ Several other pathological endothelial mechanisms contribute to fibrosis in the pathogenesis of diabetic cardiomyopathy. In the diabetic heart, the endothelium produces endothelin-1, a pro-fibrotic agent which contributes to chronic inflammation in a negative feedback loop,^163^ while endothelial-specific disruption of endothelin-1 can reduce cardiac fibrosis.^164,165^ Endothelial cells also contribute to diabetic cardiac fibrosis through the process of endothelial-to-mesenchymal transition, in which endothelial injury leads to transition to a myofibroblast-like phenotype. Endothelial-to-mesenchymal transition is widely reported in models of diabetic cardiomyopathy and is regulated by a variety of signals including miR-18a-5p/Notch2,^166^ miR-200b,^167^ Sirt6/Notch1,^168^ and kallikrein-related Peptidase 8.^169^

While endothelial cells are regarded as the primary vascular cell type involved in the pathology and pathogenesis of diabetic cardiomyopathy,^119,161^ vascular smooth muscle cells (VSMCs) and pericytes may also contribute to the disease pathogenesis. The diabetic milieu of hyperglycaemia, hyperinsulinaemia, and hyperlipidaemia has been shown to perturb VSMC contractile function and induce the pathological and highly proliferative VSMC synthetic phenotype.^170–173^ Aortic VSMCs from *db/db* mice overexpress miR-504 which inhibits contractile gene expression, promotes the synthetic phenotype, and increases proliferation and migration.^173^ Diabetic hyperglycaemia and hyperlipidaemia have been shown to directly affect the contractile function of VSMCs via inflammation-induced modulation of the microRNA (miRNA)-connexin/Rho kinase regulatory pathway,^170^ while hyperglycaemia-induced telomerase activity and nuclear factor kappa-B expression have been linked to their increased proliferation and invasion.^171,174^ In human T2D induced pluripotent stem cell (iPSC)-derived VSMCs, perturbed lipid assembly was linked to VSMC pathogenic proliferation and migration, in which overexpression of the esterase arylacetamide deacetylase was found to be cardioprotective, reducing the number of atherosclerotic lesions.^175^

Pericytes envelop the endothelial layer of small vessels and stabilize the microvasculature. Pericytes are abundant in the myocardium making up ∼5% of non-myocytes,^133^ where they secrete pro-angiogenic factors such as vascular endothelial growth factor and TGFβ, affect vascular tone, and contribute to basement membrane thickening.^176,177^ Pericyte loss is considered an early sign of microvascular diseases and is observed in diabetic nephropathy and diabetic retinopathy,^177,178^ although their role in diabetic cardiomyopathy is still unclear. Diabetic patients with end-stage HF have reduced numbers of myocardial capillaries and pericytes, accompanied by increased myocardial stiffness and loss of contractile function.^179^ Pericyte loss can occur through detachment caused by inflammatory endothelial activation^180^ or migration of the vessel,^181^ while the capacity for endothelial cells to attract pericytes to sites of neovascularization is reduced in hyperglycaemic conditions.^179^ Notably, sodium-glucose cotransporter-2 (SGLT2) inhibitors can reverse renal and retinal pericyte dysfunction induced by hyperglycaemia,^182,183^ which raises the interesting hypothesis that the beneficial effects of SGLT2 inhibitors on HF may be partially attributable to their direct actions on the cardiac microvasculature.^184^

### 5.3 The role of autonomic neurons in diabetic cardiomyopathy

The sympathetic and parasympathetic nervous systems serve to regulate bodily functions such as the respiratory rate, digestion, and heart rate.^185^ In the heart, sympathetic and parasympathetic neurons release the neurotransmitters noradrenaline and acetylcholine, respectively, to regulate cardiac function.^185,186^ Cardiac autonomic dysfunction is a recognized diabetic complication that damages the heart and increases the risk of developing arrhythmias.^187,188^ The prevalence of cardiac autonomic neuropathy (CAN) in human T2DM ranges from 25 to 75% due to the variable criteria used.^189^ Hyperglycaemia-associated injury of the autonomic nerves via ROS and toxic glycosylation products is thought to be the primary cause of CAN beginning with parasympathetic denervation followed by sympathetic denervation and relative sympathetic dominance.^190^ Recently, six phosphatidylcholine and two sphingomyelin lipid metabolites were also linked to the development of CAN in recent-onset T2DM.^191^ In several clinical studies, patients with chronic T2DM demonstrated symptoms of CAN such as high resting heart rate, decreased heart rate variability, and autonomic denervation.^131,188,192,193^

Hyperglycaemia-associated injury of sympathetic nerves represents the most common cause of sympathetic neuropathy, leading to contractile dysfunction of the cardiomyocytes.^186,194^ In animal models, investigations of hyperglycaemia-induced sympathetic dysfunction have usually employed an indirect approach by studying the cardiomyocytes rather than the sympathetic neurons. Studies have focused on the expression and function of cardiomyocyte β-adrenergic receptors, which recognize noradrenaline released from sympathetic neurons, and their downstream pathways under the conditions of hyperglycaemia and insulin resistance.^195,196^ For example, mice fed with a high-fat diet exhibit reduced cardiac sensitivity towards β-adrenergic stimulation by noradrenaline and contractile dysfunction such as increased cardiac stiffness and diastolic dysfunction.^195,196^

Autonomic neurons may also interact with cardiac vascular cells in the development of diabetic cardiomyopathy. The sympathetic co-transmitter neuropeptide Y can be found in cardiovascular neurons supplying the vasculature and cardiomyocytes.^197^ In T2DM, the expression of neuropeptide Y receptors and its activity on endothelial cells are reduced thereby blunting angiogenesis.^198^ In rat models of streptozotocin-induced diabetes, there are increased cardiac levels of both noradrenalin and acetylcholine by 8 weeks.^199,200^ Excessive levels of circulating catecholamines generate oxidative products which can induce Ca^2+^-overload in VSMCs and contribute to microvascular disease.^132^ Given that CAN in the setting of T2DM appears to be associated with the development of diabetic cardiomyopathy and increased risk of mortality,^187^ it will be of great importance to further understand the interactions between cardiac autonomic neurons and other non-myocytes in the development of diabetic cardiomyopathy.

### 5.4 The role of immune cells in diabetic cardiomyopathy

Systemic inflammation is a key feature of T2DM,^201^ and myocardial inflammation is a key process in the development of diabetic cardiomyopathy.^202–204^ In response to injury, the heart undergoes an early ‘adaptive’ inflammatory response as a remodelling and repair mechanism.^205^ However, excessive and sustained inflammation in response to a persistent stress such as diabetes, can impair this adaptive response and exacerbate cardiac injury.^206^ Diabetic conditions induce oxidative stress, the secretion of pro-inflammatory cytokines, leucocyte mobilization, and activation of NF-kB, which work in a positive feedback loop to perpetuate inflammation.^207,208^ Leucocytes are the effectors of the immune system used to maintain tissue homeostasis.^209^ In diabetic cardiomyopathy, over-activation of NF-kB increases the recruitment of leucocytes to the heart,^135,210,211^ which is associated with a higher risk of cardiovascular disease in diabetic patients.^212^ Leucocytes include neutrophils, monocytes, macrophages, lymphocytes, and platelets, among others.^213^ Neutrophils, macrophages, and lymphocytes have known roles in diabetic cardiomyopathy.^212^ Neutrophils are the first responders and constitute the first line of defence at sites of inflammation. Following cardiac injury, neutrophils polarize macrophages towards a reparative M2 phenotype.^214^ However, in diabetes, neutrophils have also been shown to secrete pro-inflammatory factors including peroxidases, cytokines, and neutrophil extracellular traps.^215,216^ Indeed, a higher neutrophil to lymphocyte ratio is an early and accurate indicator of subclinical diabetic cardiomyopathy.^217^ Extracellular glucose levels profoundly impact macrophage metabolism and identity.^218^ Diabetes-induced hyperglycaemia impairs macrophage phagocytosis,^219^ reduces the release of lysosomal enzymes,^220^ and reduces chemotactic activity,^221^ while reducing blood glucose levels has been shown to reverse these phenotypes in both rodents^222^ and humans.^223^

The classification of macrophages has recently grown to encompass a spectrum of macrophage phenotypes between the inflammatory M1-like and the reparative M2-like phenotypes, and the roles of many of these in diabetic cardiomyopathy require further elucidation.^224–227^ In diabetes, the M1-like phenotype prevails promoting a persistent low level of inflammation and insulin resistance^228,229^ while M2 macrophages reduce cardiac inflammation.^230–232^ Hyperglycaemia induces an M1 phenotype by increasing the expression of long-chain acyl-CoA synthetase-1, which promotes lipid accumulation and lipotoxicity.^233,234^ In diabetic cardiomyopathy, AGEs polarize macrophages to an M1 pro-inflammatory phenotype through a mechanism involving miR-471-3p/SIRT1,^229^ while M2 macrophages secrete macrophage mannose Receptor 1 and IL-10, reducing myocardial fibrosis and cardiomyocyte hypertrophy.^235,236^ Efferocytosis, the engulfment of apoptotic cells and debris by macrophages, is also impaired in diabetes via reduced miR-126 expression and MerTK function, thereby prolonging cardiac inflammation.^209,237^ In T2DM, cardiac inflammation and insulin resistance are also regulated by T-lymphocytes including T-helper (Th) and T regulatory (Treg) cells.^209^ Diabetes increases the number and proportion of Th1,^238^ Th17,^239^ and Th22 subtypes, which contributes to cardiac hypertrophy, fibrosis,^240^ and coronary artery disease.^241–243^ As their name suggests, Treg cells can regulate Th cells and the inflammatory response. In T2DM, Tregs can inhibit Th1, Th2, and Th17 responses to improve insulin resistance,^244^ and Treg/Th17 and Treg/Th1 ratios decline in diabetic cardiomyopathy.^238,245^ Balancing pro-inflammatory (Th1 and Th17) and regulatory (Treg) T cells may be a viable therapeutic approach to manage or prevent chronic cardiac inflammation.

In their interactions with other cardiac non-myocytes, cardiac immune cells can signal to cardiac fibroblasts to influence fibrosis and contribute to cardiac remodelling in diabetic cardiomyopathy. Inflammatory signals such as TNFα, IL-1β, and TGFβ can act directly on cardiac fibroblasts to promote fibrosis.^165^ For example, TNFα stimulates cardiac fibroblast proliferation and collagen production via WISP1,^246^ while IL-1β induces cardiac fibroblasts to release insulin-like growth factor-1 via STAT3 signalling to promote cardiomyocyte hypertrophy.^247^ TGFβ, the primary pro-fibrotic cardiac cytokine secreted by macrophages, T-cells, and fibroblasts themselves, is responsible for fibroblast activation.^95,204^ Finally, injury-site macrophages can transition into fibroblast-like cells with profound ramifications for our understanding of the inflammation-healing-axis.^248^ Macrophage-to-myofibroblast transition has been observed in myocardial infarction,^249^ diabetic nephropathy,^250^ and a T2DM model of dermal wound healing,^251^ but not yet reported in the context of diabetic cardiomyopathy. Notably, following myocardial infarction, cardiac macrophages can transition to a myofibroblast-like cell able to synthesize immature forms of collagen and elastin.^249^ However, these macrophage-derived myofibroblast-like cells do not respond to typical pro-fibrotic signals such as aldosterone, angiotensin II, or hypoxia, suggesting a different underlying mechanism and that new approach may be needed to combat their fibrotic activity.^249^ Interestingly, in the T2DM model of dermal wound healing, there was a reduced transition rate of myeloid cells to myofibroblasts and reduced wound collagen and skin stiffness which was rescuable via a miRNA-21 mimetic.^251^ In the setting of diabetic cardiomyopathy, this may explain the prolonged retention of cardiac inflammatory macrophages which are unable to transition into their intended fibroblast state.

## 6. Extracellular vesicles mediate cellular and tissue crosstalk

In addition to soluble factors, intercellular transfer of extracellular vesicles (EVs) has emerged as an important paracrine mechanism for cells to communicate with nearby and distant cells. EVs are nano-sized lipid membrane-bound particles endogenously released from various cell types in the body. These nano-sized vesicles contain a range of bio-reactive materials (proteins, lipids, metabolites) and genetic material (DNA, messenger RNA, and non-coding RNAs) that are unique to each cell type and altered in disease states like T2DM. EVs are involved in many pathophysiological processes including cardiometabolic diseases.^252,253^ The contribution of EVs to the progression of diabetic cardiomyopathy has been comprehensively reviewed by others^254,255^ and will be briefly discussed here.

In T2DM patients, circulating endothelial cell-derived EVs are elevated, correlating with an increased risk of diabetes-related vascular complications and atherogenesis.^256–258^ Plasma EVs isolated from T2DM patients show aberrant miRNA expression profiles,^259,260^ while intercellular crosstalk among cardiovascular system-related cells via secreted EVs has been implicated in various pathological processes including impaired angiogenesis, inflammation, myocardial hypertrophy, and fibrosis, all of which can lead to diabetic cardiomyopathy.^255^ For example, EVs isolated from diabetic subjects contain higher levels of anti-angiogenic miR-320, and lower levels of angiogenic miR-126 and vascular endothelial growth factor receptor-2.^261–264^ Transfer of miR-320 from cardiomyocyte-derived EVs to endothelial cells results in impaired angiogenesis.^261^ Similarly, hyperglycaemia has been shown to up-regulate anti-angiogenic miR-503 expression in endothelial cell-derived EVs and functional transfer of miR-503 negatively affects pericyte migration and proliferation.^265^ In addition to miRNAs, EVs isolated from the plasma of diabetic subjects were shown to contain high levels of Arginase 1, which can cause vascular dysfunction when taken up by endothelial cells.^266^ Transfer of miR-21-3p-enriched EVs from cardiac fibroblasts to cardiomyocytes has been reported to induce cardiomyocyte hypertrophy through silencing of sorbin and SH3 domain-containing protein 2 and PDZ and LIM Domain 5,^267^ although whether this paracrine miRNA crosstalk is exacerbated by diabetes remains to be determined.

In addition to miRNAs, other non-coding RNAs such as long non-coding RNAs (lncRNAs) and circular RNAs (circRNAs) are also known to play a significant role in the disease development and progression of diabetic cardiomyopathy, as reviewed comprehensively elsewhere.^268–270^ For example, lncRNA H19 expression was down-regulated in the myocardium of diabetic rats and high glucose-treated cardiomyocytes, and was functionally associated with cardiomyocyte apoptosis, oxidative stress, and cardiac muscle dysfunction.^271^ In another study, lncRNA Kcnq1ot1 expression was found to be elevated in the myocardium of diabetic mice and high glucose-treated cardiomyocytes and cardiac fibroblasts, and was associated with fibrosis, inflammation, and pyroptosis.^272,273^ In addition to the myocardium, several dysregulated lncRNAs, such as E330013P06, Dnm3os (dynamin3 opposite strand), and Diabetes Regulated anti-inflammatory RNA, have been found in the immune cells of diabetic subjects.^274–276^ Up-regulation of LncRNA_E330013P06 in diabetic mouse macrophages has been shown to induce expression of pro-inflammatory genes and promote foam cell formation, which are major risk factors of atherosclerosis.^275^ Other lncRNAs that have been implicated in the pathogenesis of diabetic cardiomyopathy include HOTAIR,^277^ MALAT1,^278^ MIAT,^279,280^ CRNDE,^281^ and TUG1,^282^ among others.^269,274^ In regard to circRNAs, up-regulation of circRNA_000203 has been found in diabetic mouse myocardium and was associated with increased expression of pro-fibrotic genes in cardiac fibroblasts.^283^ Similarly, circRNA_010567 expression was found to be up-regulated in murine diabetic myocardium and implicated in myocardial fibrosis by sponging miR-141, which directly targets pro-fibrotic TGF-β1.^284^

Most studies have only reported the roles of lncRNAs and circRNAs as intracellular signalling regulators of pathological mechanisms of diabetic cardiomyopathy. Indeed, there are only limited studies describing the intercellular transfer of these non-coding RNAs. Over 3350 circulating lncRNAs were shown to be dysregulated in the plasma of Type 2 diabetic mouse model with impaired cardiac function.^285^ In that study, the Top 5 networked lncRNAs XLOC015617, AK035192, Gm10435, TCR-α chain, and MouselincRNA0135 were involved in the development and motion of myofilaments, the immune response, and apoptosis.^285^ In serum from diabetic patients, lncRNA expression of TINCR^286^ and NKILA^287^ can be used to predict diabetic cardiomyopathy up to 6 months before a clinical diagnosis,^287^ indicating that circulating lncRNAs may be easily accessible serum biomarkers as well as therapeutic targets.

EVs also play a role in inter-organ crosstalk. Circulating EVs generated remotely by non-cardiac tissues in the diabetic milieu, have also been implicated in the development and progression of diabetes complications.^288,289^ EVs from visceral adipose tissues of high-fat diet-induced obese mice have been shown to reprogramme macrophages into the pro-inflammatory M1 phenotype and induce chronic atherosclerotic inflammation, a risk factor of diabetic cardiomyopathy, when delivered intravenously into hyperlipidemic apolipoprotein E-deficient mice.^290^ Similarly, EVs isolated from 3T3-L1 adipocytes subjected to simulated insulin resistance were enriched with sonic hedgehog protein and induced pro-inflammatory M1 polarization of macrophages via the Patched(Ptch)/PI3K signalling pathway.^291^ Interestingly, EVs from adipose tissue macrophages of obese mice overexpressed miR-155, which targets peroxisomal proliferator-associated receptor γ and led to glucose intolerance and insulin resistance in other tissues such as muscle and liver when administered into lean mice.^292^

In the light of these findings, the cargo of EVs could potentially be engineered to deliver beneficial biomolecules as a new avenue for treating diabetes complications.^289,293^ Indeed, using streptozotocin-induced diabetic mice with cardiac-specific overexpression of Hsp20, Wang *et al*.^294^ have demonstrated that cardiomyocyte-specific EVs carrying overexpressed Hsp20 beneficially attenuate streptozotocin-induced cardiac dysfunction and adverse remodelling. Interestingly, overexpression of Hsp20 also altered the cargo of cardiomyocyte EVs to contain cytoprotective proteins such as phosphorylated-Akt, superoxide dismutase, and survivin.^294^

## 7. Preclinical models of diabetic cardiomyopathy

### 7.1 Animal models

**Figure 3:**
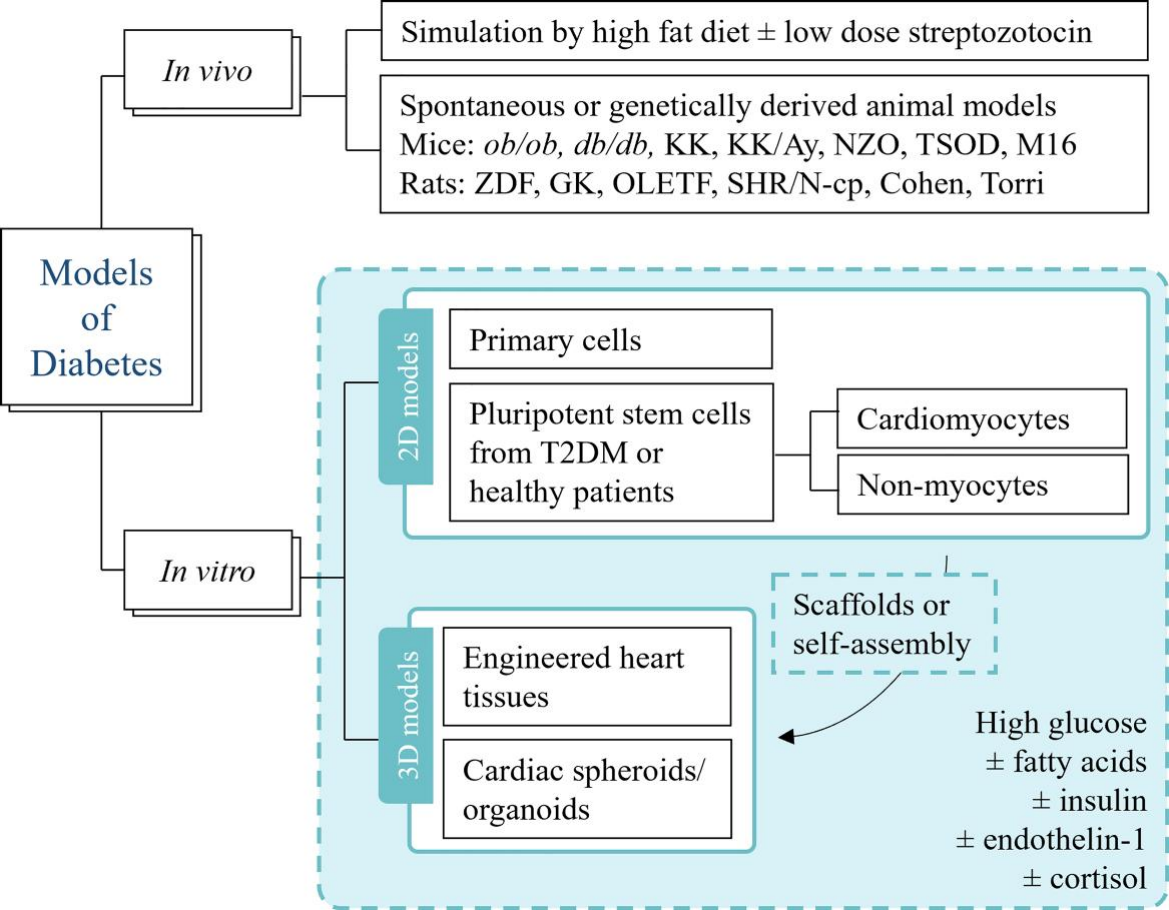
**An overview of current preclinical models of Type 2 diabetes.** *ob/ob*, leptin deficient mouse (obese); *db/db*, leptin resistant mouse (obese); KK, Kuo-Kondo mouse (obese); KK/A^y^, yellow KK mouse (obese); NZO, New Zealand obese mouse (obese); TSOD, Tsumara Suzuki obese diabetes mouse (obese); M16 mouse (obese); ZDF, Zucker diabetic fatty rat; GK, Goto-Kakizaki rat; OLETF, Otsuka Long-Evans Tokushima Fat rat; SHR/N-cp, spontaneously hypertensive rat/NIH-corpulent rat (obese); Cohen, Cohen diabetic rat (non-obese); Torri, Torri rat (non-obese); T2DM, Type 2 diabetes mellitus.

Our understanding of the pathophysiology and identification of therapeutic targets for diabetic cardiomyopathy has relied predominantly on *in vivo* and *in vitro* animal models (*Figure 3*), in particular, T2DM murine models.^295,296^ In murine models, T2DM can be modelled using a high-fat diet with or without injection of streptozotocin, which is toxic to the β-cells.^296^ The combination of high-fat diet and streptozotocin allows the animal to mimic the destruction of some β-cell islets, a hallmark of late-stage T2DM.^296,297^ Animals fed with high-fat diet (50–60% kcal fat) often develop cardiac hypertrophy, cardiac fibrosis, and contractile dysfunction. Despite exhibiting many of the cardiac phenotypes similar to patients with diabetic cardiomyopathy, high-fat diet animal models display significant variation in their development of diabetic cardiomyopathy due to a range of food compositions, length of high-fat feeding, and the age of the animal at treatment onset.^298^

Genetic manipulation has given rise to transgenic small animals that can spontaneously develop hyperglycaemia, insulin resistance, and cardiac abnormalities resembling diabetic cardiomyopathy.^296^ For example, *ob/ob* and *db/db* mice are based on leptin deficiency or resistance, respectively, and are commonly used for the study of diabetic cardiomyopathy.^296,299,300^ Similarly, there are transgenic rats such as Zucker diabetic fatty rats and Goto-Kakizaki inbred rats.^295^ Zucker diabetic fatty rats develop T2DM because of a non-functional leptin receptor, whereas Goto-Kakizaki rats spontaneously develop diabetes as a consequence of inbred between Wistar rats which displayed genetic susceptibility to develop high blood glucose.^162,295,301^ However, transgenic animal models can suffer from either too mild or too severe phenotypes, premature death, or highly variable data arising from the underlying mutations, all of which can affect their translatability to the human disease setting.^302^

Rodent models are favourable tools to study T2DM and cardiovascular disease due to (i) their short breeding cycle, (ii) ease of genetic manipulation, and (iii) the relatively high degree of genetic homology between mice and humans.^296^ However, there are fundamental physiological differences between animals and humans. For example, the heart rate of a mouse is ∼600 beats per minute, which is in stark contrast to ∼60 beats per minute in the human.^303^ There are also differences in the cardiac physiology arising from the different machinery in calcium handling, as well as a different expression of contractile protein isoforms and ion channels.^303^ Thus, there will inevitably be results obtained from animal models that cannot be translated to humans. For example, dipeptidyl-peptidase 4, a potential therapeutic for diabetic cardiomyopathy, failed to yield positive outcomes in clinical trials despite promising results shown in preclinical animal models.^5,304,305^

### 7.2 Human cell-based models of diabetes

Isolated primary adult cardiomyocytes are the closest to native adult human cardiomyocytes in terms of morphology, electrophysiology, metabolism, as well as proteomic and transcriptomic profiles. Primary human cardiovascular cells cultured in hyperglycaemic and hyperlipidaemic conditions to simulate T2DM, or those isolated from T2DM patients, would be the ideal human-specific models to study diabetic cardiomyopathy and to help streamline the translation of findings that are safe and effective in humans.^205^ However, studies on primary human cardiomyocytes are lacking in the literature, due to the scarcity of viable human heart tissue, and the technical difficulties in isolating and maintaining primary adult cardiomyocytes.^306^ These limitations warrant the establishment of alternative pre-clinical human cardiac models for mechanistic studies, as well as for drug discovery, which can be addressed with human PSCs.^307,308^ Human PSCs can self-renew indefinitely, to provide an inexhaustible source of human cells for studying human diseases and drug screening. PSCs can also differentiate into all derivatives of the three germ layers, including *bona fide* cardiovascular cells, which can be cultured for extended periods of time allowing for chronic treatment of test compounds.^307,309,310^ Importantly, unlike primary cardiomyocytes isolated from biopsies that are a snapshot in time, cardiovascular cells derived from PSCs provide an opportunity to examine the early stages of diseases and can be used to study the progressive pathogenic mechanisms of diseases.

There are two main types of PSCs, embryonic stem cells, and iPSCs. Human embryonic stem cells are derived from the undifferentiated inner cell mass of a human embryo at the blastocyst stage and were first isolated in 1998.^311^ However, the applications of human embryonic stem cells are often restricted by the ethical concerns surrounding the destruction of a human embryo, the difficulty in obtaining embryonic stem cells with specific disease phenotypes, and they are usually not genetically matched with patients for transplantation.^307^ The breakthrough offered by iPSCs has largely overcome these limitations. Following their success in generating the first iPSCs from mouse fibroblasts, Yamanaka and co-workers^312^ established human iPSCs from human fibroblasts through lentivirus-mediated transduction of pluripotency factors OCT3/4, SOX2, KLF4, and c-MYC. Importantly, iPSCs have the same genetic makeup as the donor cells from which they were generated, making them a promising cell source for personalized medicine. Since their discovery, human iPSCs have been widely employed to model different diseases including diabetic cardiomyopathy.^307^ Significantly, iPSCs generated from different patients with diverse generic backgrounds will capture clinical heterogeneity and enable disease modelling and drug testing at the population level. This idea was made possible through the establishment of HLA haplobanks with clinical-grade iPSC lines from donors covering the worldwide population by several organizations such as the Global Alliance for iPSC Therapies (GAiT).^313–315^

Cardiomyocytes derived from human iPSCs have been employed to model diabetic cardiomyopathy^316–318^ (*Table 1*). Similar to findings in animal models, cardiomyocytes derived from human iPSCs cultured in high glucose conditions exhibit an appropriate disease phenotype such as increased expression of hypertrophic markers and pro-inflammatory factors, accumulation of intracellular lipids, and reduced contractility.^316–318^ Although cardiomyocytes derived from patient iPSCs have conventionally been used to model a variety of hereditary monogenic cardiac disorders,^327,328^ they are increasingly being appreciated for their ability to faithfully capture the cardiac pathophysiology of various non-hereditary lifestyle-related cardiac diseases and cardiac diseases with polygenic risk variants like T2DM.^329^ Indeed, cardiomyocytes derived from iPSCs generated from T2DM patients have been shown to recapitulate known cardiomyopathic phenotypes including cellular hypertrophy, impaired contractility, calcium handling abnormalities, metabolic dysregulation, and abnormal TGF-β signalling in the absence of diabetic culture conditions.^308,316^

**Table 1:** Modeling diabetic cardiomyopathy and vasculopathy with human PSCs

Cell composition	Simulated diabetes conditions	Phenotypes	References
2D models			
ȃCMs	10 mM glucose, 10 nM endothelin-1, and 1 µM cortisol for 2 days	•Cellular hypertrophy •Increased Troponin I and FABP3 proteins secretion •Loss of sarcomeric integrity •Reduced frequency of calcium transients •Increased frequency of irregular beat rate •Increased intracellular peroxidized lipid	Drawnel *et al.*^316^
ȃCMs	Derived from T2DM patients’ iPSCs	•Loss of sarcomeric integrity •Reduced frequency of calcium transients •Increased frequency of irregular beat rate •Increased intracellular peroxidized lipid	Drawnel *et al.*^316^
ȃCMs	25 mM glucose for 1 day	•Increased superoxide production •Increased mitochondrial membrane potential •Increased mitochondrial fission	Canfield *et al*.^319^
ȃCMs	33 mM glucose	•Reduced glycolytic activity and mitochondrial respiration	Prakoso *et al*.^320^
ȃCMs	10 mM glucose, 10 µg/mL insulin, 0.2 mM palmitic acid, 10 nM endothelin-1, and 1 µM cortisol for 3 days	•Increased intracellular lipid droplets and ceramide levels •Increased mitochondrial superoxide levels •Reduced oxygen consumption, mitochondrial β-oxidation and respiratory capacity •Increased mitophagy •Loss of sarcomeric integrity •Reduced beating rate •Increased frequency of irregular calcium transients	Bekhite *et al*.^321^
ȃCMs	Derived from T2DM patients’ iPSCs	•Increased intracellular lipid droplets •Cellular hypertrophy •Increased sensitivity to glucolipotoxicity •Reduced mitochondrial membrane potential •Increase ROS •Reduced oxygen consumption and ATP production •Increased frequency of irregular beat rate •Increased frequency of irregular calcium transients	Tang *et al*.^308^
ȃCMs	10 mM glucose, 10 nM endothelin-1, and 1 µM cortisol for 3 days	•Cellular hypertrophy	Tang *et al*.^308^
ȃCMs	22 mM glucose for 14 days	•Cellular hypertrophy •Reduced contractility •Increased amplitude of calcium transient •Reduced glycolytic capacity	Ng *et al*.^317^
ȃEndothelial cells	Derived from T2DM patients’ iPSCs	•Reduced angiogenic potential •Reduced NO production •Increased endothelin-1 secretion •Disrupted glycine homeostasis •Reduced growth rates and increased cell senescence •Reduced mitochondrial membrane potential and ATP production	Su *et al*.^322^
ȃEndothelial cells	33 mM glucose and 10 nM endothelin-1 for 3 days	•Reduced angiogenic potential •Increased ROS •Reduced ATP levels •Reduced autophagy •Increased mitochondria fragmentation •Increased susceptibility to simulated ischaemia-reperfusion injury	Ong *et al*.^323^
3D models			
ȃCMs	12 mM glucose for 7 days	•Impaired self-assemble into compact microtissue with thicker fibres •Shorter duration of calcium transient and larger amplitudes of calcium transient when subjected to electrical stimulation •Increased beating rate	Balistreri *et al*.^324^
ȃCMs, epicardial cells, endocardial cells, endothelial cells, cardiac fibroblasts.	11.1 mM glucose and 1.14 nM insulin for 2 weeks	•Increased in organoid size •Higher frequency of irregular action potential •Reduced oxygen consumption and increased glycolysis •Increased lipid droplet •Myocyte structural defects	Lewis-Israeli *et al*.^325^
ȃEndothelial cells, pericytes	75 mM glucose ± 1 ng/mL TNF and 1 ng/mL IL-6 for 3 weeks	•Reduced ratio of endothelial cells to pericytes •Increased vascular membrane ECM (collagen type IV, fibronectin, laminin, and perlecan) •Thickening and splitting of the basement membrane layer	Wimmer *et al*.^326^

CMs, cardiomyocytes; ECM, extracellular matrix.

### 7.3 Multicellular human organoid models

**Figure 4:**
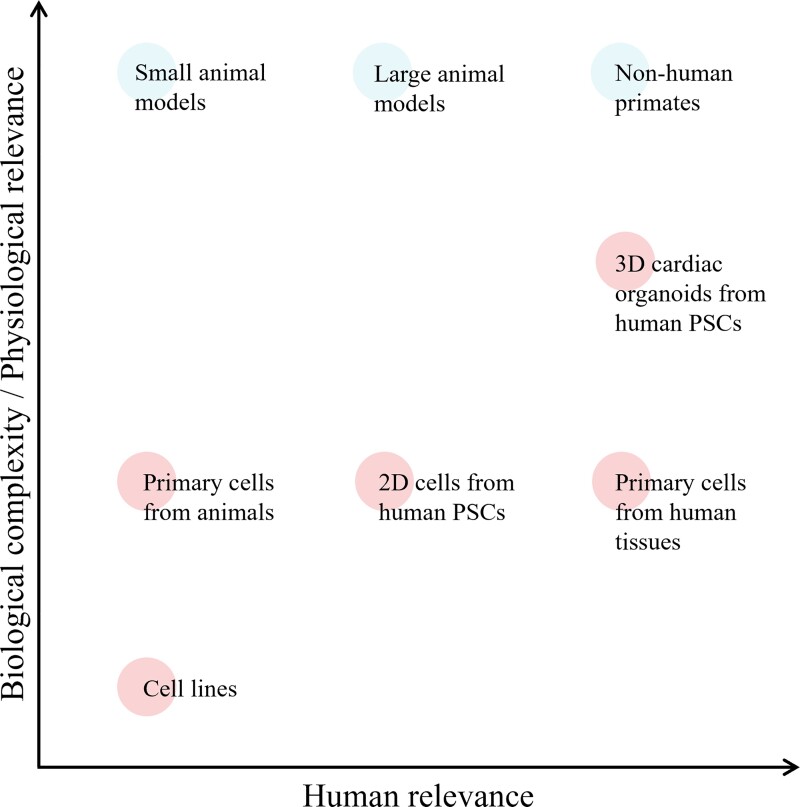
**The physiological and human relevance of preclinical disease models.** The development of effective therapies for patients is largely impeded by the lack of reliable models with strong biological relevance to human disease. *In vivo* (blue circles) animal models, especially small animal models, have predominantly been employed for their physiological complexity at the expense of biological relevance to the human. Although primary cells from human cardiac tissues represent the most ideal cell type to model human disease, their availability and accessibility are limited. The establishment of 3D cardiac organoids from human PSCs has revolutionized human disease modelling, providing a mimicry of human heart physiology *in vitro* (red circles). Cardiac organoids provide enhanced biological complexity such as a 3D microenvironment allowing interaction between cardiomyocytes and non-myocytes compared with the conventional *in vitro* culture, which is generally monocellular 2D culture.

Unlike the native heart, 2D cell culture models do not fully recapitulate complex cellular interactions between different cell types under physiological and pathophysiological conditions such as diabetic cardiomyopathy (*Figure 4*).^307^ The advent of 3D cell culture systems, such as self-organizing cardiac organoids, has addressed this limitation to some extent. A cardiac organoid is a 3D tissue mimicry that can recreate the native microenvironment and tissue architecture as well as model various pathophysiological processes *in vitro*.^330–335^ In recent years, several studies had established human iPSC-derived cardiac organoids and microtissues consisting of cardiomyocytes and non-myocyte cell populations, mainly fibroblasts and endothelial cells.^333,336,337^ The possibility to derive all cardiac cell types from a single human iPSC source will permit isogenic disease modelling and pave the way for personalized medicine. However, further developments to incorporate other essential non-myocyte cell populations such as autonomic neurons, VSMCs, pericytes, and immune cells are needed to accurately model the cellular heterogeneity of native heart tissue and the tissue response to disease stimuli. Recent studies showing functional co-culture of human iPSC-derived sympathetic neurons and cardiomyocytes in a 2D culture system^338,339^ raise the possibility of establishing an innervated 3D cardiac organoid model. These multi-cell type models of the heart can be expected to play a huge role in the early stages of drug development where the prediction of cardiac toxicity will be improved, allowing a more efficient transition from preclinical to clinical studies.^330^ For example, a model of diabetic vasculopathy was recently described, involving a self-organizing 3D human blood vessel organoid model capable of modelling diabetic vasculopathy when cultured in high glucose media or exposed *in vivo* to a diabetic milieu in mice.^326^ The blood vessel organoids generated through directed differentiation of human PSCs consist of networks of CD31^+^ endothelial cells surrounded by pericytes, mesenchymal stem-like cells, and CD45^+^ haematopoietic cells. Using this advanced pre-clinical human 3D model, the authors successfully identified NOTCH3 and its ligand DLL4 as a new potential therapeutic target for diabetic microvasculopathy.^326^

Currently, cardiac organoid technology is presented with challenges including insufficient robustness and reliability for mimicking complex multicellular microenvironments, reproducibility of protocols with appropriate quality controls, and the precise ratio of cardiomyocyte and other non-myocytes to achieve a robust model.^330^ In addition to the intercellular crosstalk within the heart tissue, inter-organ crosstalk is also regarded as highly relevant to the pathophysiology of systemic diseases like diabetes. In this regard, the development of organ-on-a-chip technologies has allowed studies of crosstalk between organs in disease development and progression.^340–342^ These organ-on-a-chip platforms allow simultaneous culture of multiple bioengineered organoids or microtissues from different organs in a closed circulatory perfusion microfluidic device under a controlled environment of medium flow between different ‘organ’ compartments and local electromechanical properties.^340–342^ The evolution of multi-organ on a chip approaches may provide superior *in vitro* systems to study the dynamic biochemical interactions and responses of cells from different organs in diabetic conditions for drug and therapy development.

## 8. Therapies for diabetic cardiomyopathy

Treatments for diabetic cardiomyopathy have largely focused on managing the symptoms of diabetes, such as controlling glycaemic control, lowering lipid levels, and managing oxidative stress.^5^ However, glucose-lowering treatments such as insulin sensitisers,^343^ glucagon-like peptide (GLP1) agonists,^344^ sulfonylureas, and some dipeptidyl peptidase-4 inhibitors,^345^ have had limited clinical success preventing HF and can even increase the risk of HF. Metformin, a first-line treatment for T2DM, has been contraindicated for patients with HF due to the possible risk of lactic acidosis.^346^ However, recent pre-clinical and clinical data suggest the risk of lactic acidosis may no higher for metformin than for any other anti-hyperglycaemic drug.^347,348^ Most pre-clinical studies have emphasized intra-cardiomyocyte signalling pathways and overlooked the crosstalk between cardiomyocytes and other supporting cells. The interaction between cardiomyocytes and other resident cardiac non-myocytes such as endothelial cells and cardiac fibroblast has gained increasing attention and it is undoubtedly important for maintaining cardiac physiology and driving pathological processes.^135^ For instance, endothelial dysfunction can result in hyperinsulinaemia, glucose intolerance, and insulin resistance in the mouse following the inducible endothelial-specific knockout of bone morphogenetic protein-binding endothelial regulator through its interaction with the cholesterol transporter Niemann-Pick C1.^349^ This suggests an active endocrine role for the endothelium in regulating glucose homeostasis and a potential basis for cardiovascular disease in T2DM.

### 8.1 SGLT2 inhibitors as promising therapeutics for diabetic cardiomyopathy

Perhaps the most exciting and promising news in diabetic cardiomyopathy and HF more generally, is the recent advent of the SGLT2 inhibitors as a therapy for HFpEF.^350^ Initially promoted solely as a class of antidiabetic drugs, SGLT2 inhibitors improve glycaemic control, reduce body mass, and lower blood pressure.^351^ However, over several clinical trials, the SGLT2 inhibitors dapagliflozin,^352^ empagliflozin,^353^ canagliflozin,^354^ and others, were shown to prevent HF and reduce hospitalization and cardiovascular death in T2DM patients with HFrEF. A recent hallmark study, the Empagliflozin Outcome Trial in Patients with Chronic HFpEF (EMPEROR-Preserved), showed that empagliflozin reduced the risk of cardiovascular death or hospitalization for HF in both diabetic and non-diabetic patients with HFpEF.^350^ Significantly, this makes SGLT2 inhibitors the first class of medications to confer benefits across both HFrEF and HFpEF, and a promising therapy for treating diabetic cardiomyopathy.

### 8.2 The cardioprotective effect of SGLT2 inhibitors

The cardioprotective effects by SGLT2 inhibitors are known to be independent of glycaemic control, or conventional risk factors such as body mass and blood pressure.^355,356^ It has been widely reported that SGLT2 is expressed almost exclusively in the proximal tubular cells of the kidney where it facilitates glucose reabsorption.^357,358^ Numerous hypotheses for how SGLT2 inhibitors confer cardioprotection have been proposed. These include reduced cardiac remodelling, reduced plasma volume, improved diastolic function and Ca^2+^ and Na^2+^ homeostasis, reduced oxidative stress and inflammation, increased metabolism of ketone bodies, and increased autophagy, which have been recently reviewed.^359^ Undoubtedly, the mechanisms underlying the cardioprotective effect of SGLT2 inhibitors remain unclear, and made more difficult to explain by the apparent absence of SGLT2 in human heart tissue.^360^ Interestingly, several lines of evidence point to SGLT2 expression in different vascular cell types including bovine retinal pericytes,^183^ human umbilical vein endothelial cells,^361^ and mouse aorta and aortic endothelial cells.^362^ While at lower levels than in the kidney, there are reports of SGLT2 expression in aortic VSMCs isolated from human^363^ and rat.^364^ Patient-derived epicardial adipose tissue and stromal vascular cells also express SGLT2, which was shown to promote glucose uptake in response to dapagliflozin.^365^ These studies suggest another plausible mechanism of SLGT2 inhibitor-mediated cardioprotection via either direct or indirect actions on the cardiac non-myocyte populations and extra-cardiac tissues.

Recent reports document beneficial *in vivo* and *in vitro* effects of SGLT2 inhibitors on VSMC^363,364^ and endothelial function^362,366–369^ in both diabetes and non-diabetes. Endothelial function is improved by SGLT2 inhibitor therapy in patients with either uncontrolled T2DM^367^ or those with T2DM and chronic HF.^368^ In mouse and rat models of T2DM, SGLT2 inhibitors have been shown to rescue endothelial function,^362^ improve impaired endothelium-dependent vasodilation by increasing eNOS activity,^369^ and reduce neointima formation following vascular injury when used in combination with dipeptidyl peptidase-4.^364^ In non-diabetic settings, canagliflozin has been shown to preserve the integrity of cellular junctions in human endothelial cells exposed to septic plasma via an α1AMPK-dependent pathway,^370^ while empagliflozin prevents VSMC transition towards a synthetic phenotype, reducing proliferation and migration following IL-7A induced oxidative stress and inflammation.^363^ Endothelial cells can undergo endothelial-to-mesenchymal transition and contribute to fibrosis in diabetic cardiomyopathy.^371^ Both dapagliflozin and metformin have been shown to reduce cardiac fibrosis and endothelial-to-mesenchymal transition in peripheral endothelial cells in a mouse model of T2DM induced by high-fat diet feeding combined with streptozocin treatment.^372^ Human umbilical vein endothelial cells exposed to hyperglycaemia also undergo endothelial-to-mesenchymal transition and induce excessive extracellular collagen deposition, which can be reversed with dapagliflozin through AMPKα-mediated TGFβ signalling.^372^

SGLT2 inhibitors might also exert cardioprotection by regulating the immune response. Following myocardial infarction, SGLT2 inhibitors increase cardiac M2 macrophage numbers and levels of IL-10, a potent anti-inflammatory cytokine.^373^ Serum levels of IL-10 also increase following dapagliflozin treatment in T2DM patients.^374^ In addition to the immune response, evidence also points to an effect of SGLT2 inhibitors on the autonomic nervous system. T2DM patients treated with SGLT2 inhibitors report a lower incidence of ventricular arrhythmias^375^ and atrial flutter.^376^ Traditionally, diuretics-like SGLT2 inhibitors increase sympathetic tone.^377^ However, treatment with SGLT2 inhibitors does not increase sympathetic tone,^378^ suggesting that they may act on the sympathetic nervous system to reduce hyperactivity.^379,380^ Indeed, dapagliflozin has been shown to attenuate the increase in cardiac and renal tyrosine hydroxylase positive nerve fibres observed in a mouse model of T2DM induced by high-fat diet.^381^ However, the expression of SGLT2 in human autonomic neurons remains to be determined. Collectively, these studies highlight the possible effects of SGLT2 inhibitors on the cardiac non-myocytes, plausibly by systemic actions, which could be harnessed as a specific therapy for diabetic cardiomyopathy.

## 9. Conclusion

The pathogenesis of diabetic cardiomyopathy involves a combination of abnormal extracellular matrix deposition, metabolic perturbations, oxidative stress, and inflammation, which lead to pathogenic cardiac remodelling and dysfunction. While pro-inflammatory cytokines, AGEs and ROS drive maladaptive changes to the cardiomyocytes, the pathogenesis of diabetic cardiomyopathy cannot be explained by these factors alone. Cardiac fibroblasts, the coronary vasculature, the autonomic nervous system, and immune cells regulate electrical, chemical, and biomechanical signalling in the heart, maintain cardiac homeostasis, and in diabetic conditions, contribute to the pathogenesis of diabetic cardiomyopathy. The involvement of non-myocytes in the pathogenesis and pathology of diabetic cardiomyopathy should not be viewed as an impediment, but rather as an opportunity to leverage our existing models and treatments for the development of new and effective therapeutic strategies.

## Authors’ contributions

J.G.L. and S.Y.L. conceived the idea for the article; R.J.P., J.G.L., and S.Y.L. performed the literature search and data synthesis; R.J.P. prepared the original draft; R.J.P., J.G.L., S.Y.L., R.H.R., and D.J.H. critically revised the work.

## Data Availability

No new data were generated or analysed in support of this research.
